# Epidemiology and Trends in Cartilage Surgery of the Foot and Ankle in Germany: An Analysis of National Healthcare Billing and Reporting Data from 2006 to 2020

**DOI:** 10.3390/medicina59071256

**Published:** 2023-07-06

**Authors:** Annette Eidmann, Tizian Heinz, Jan Oberfeld, Manuel Weißenberger, Maximilian Rudert, Ioannis Stratos

**Affiliations:** Department of Orthopaedic Surgery, University of Wuerzburg, Koenig-Ludwig-Haus, Brettreichstrasse 11, 97074 Wuerzburg, Germany; t-heinz-klh@uni-wuerzburg.de (T.H.); drjanoberfeld@gmail.com (J.O.); manuel.weissenberger@googlemail.com (M.W.); m-rudert.klh@uni-wuerzburg.de (M.R.); i-stratos.klh@uni-wuerzburg.de (I.S.)

**Keywords:** cartilage surgery, foot and ankle procedures, epidemiological analysis, regenerative therapies, age distribution

## Abstract

*Background and objectives*: Cartilage surgery constitutes a standard intervention in foot and ankle procedures. Currently, there is a lack of epidemiological data on its frequency, age distribution, and surgical options for cartilage surgery. This study aimed to investigate the current landscape of cartilage surgery in Germany and identify the most common procedures from an epidemiological standpoint. *Materials and methods*: Medical billing and reporting data from the Federal Statistical Office of Germany, encompassing the period 2006–2020, was examined, including all foot and ankle cartilage surgical procedures (summarized under OPS codes 5-812 and 5-801). The dataset incorporated information on the affected joint, patient age and sex, and surgery type. Each surgical procedure was categorized as “debridement”, “regeneration” or “refixation”. Linear and nonlinear regression analyses were employed, with a statistical significance threshold of 0.05. *Results*: From the total of 136,501 procedures conducted during the study period, the most frequently performed interventions were microfracture (58,252) and chondroplasty (56,135), and thus, debridement procedures were in the leading position. The use of acellular membranes was the most used regenerative technique (n = 11,414). At the ankle joint, interventions were mostly arthroscopic and in men, while foot cartilage surgeries were preferably performed via open surgery and mostly in women. Age distribution analysis revealed two primary peaks: the first in the 20–25-year-old group (ankle and foot) and the second in the 45–50-year-old group (ankle) and 55–60-year-old group (foot). Refixation and regenerative procedures were more frequent among younger individuals, while debriding procedures were more frequent among older individuals. Regenerative procedures, particularly in the ankle, significantly increased over time. *Conclusions*: Cartilage surgery of the foot and ankle was common, with two primary age groups predominantly affected. Notably, recent years have witnessed a considerable rise in cartilage regenerative procedures.

## 1. Introduction

Cartilage defects are prevalent pathologies in the foot and particularly the ankle, yet epidemiological data remain scarce. Ankle lesions primarily consist of osteochondrosis dissecans in adolescent patients or osteochondral lesions, which are believed to result from direct trauma or repetitive microtrauma [1,2]. Foot joint lesions often stem from primary or secondary arthrosis, potentially caused by malalignment.

**Figure 1:**
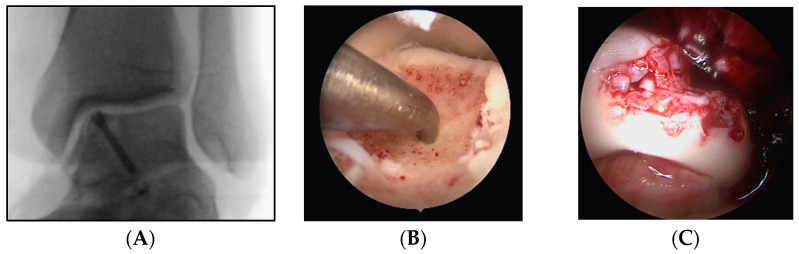
Three intraoperative images of different patients with osteochondral defects of the upper ankle joint. Reattachment of an osteochondral defect using a double-threaded screw (**A**), microfracturing of an osteochondral defect (**B**), and minced cartilage repair (placement of cartilage chips as a substitute for damaged cartilage (**C**) of the medial shoulder of the talus.

Numerous therapeutic methods have been developed to address symptomatic cartilage lesions in the foot and ankle, which often necessitate surgical intervention. Depending on factors such as patient age, activity level, and defect morphology, treatment for chondral lesions may have a regenerative or palliative intention. Regenerative therapeutic approaches include cartilage transplantation, in vitro tissue culture implantation, and subchondral bone opening with or without acellular implant insertion and homologous whole blood or component enrichment (Figure 1). Palliative methods, on the other hand, encompass procedures such as diseased cartilage debridement [1,3]. Surgical interventions can be performed arthroscopically or through open surgery, contingent upon the procedure and affected joint.

Arthroscopy of the ankle, first described in the 1930s [4], gained widespread popularity following the work of Watanabe [5] and Chen [6] in the 1970s. Presently, ankle joint arthroscopy ranks as the third most common arthroscopic procedure, with its popularity and availability continuing to rise [7]. The arthroscopy of smaller joints, such as the first metatarsophalangeal joint, has further expanded the range of therapeutic techniques in recent years [8].

Despite the high prevalence of cartilage lesions in the foot and ankle and the widespread application of their treatments, limited evidence exists regarding the choice of therapeutic procedures [9]. Additionally, it remains unclear whether specific procedures should be performed arthroscopically or via arthrotomy. In the absence of definitive guidelines, the frequency of performed procedures may offer insight into the therapies that yield favorable outcomes based on surgeons’ subjective experiences. Consequently, this study aimed to examine the current state of cartilage surgery for the foot and ankle in Germany, with a particular emphasis on the diverse therapeutic procedures within the context of epidemiological data and their evolution over time.

## 2. Materials and Methods

In the research presented in this paper, we conducted an analysis of 136,501 surgical procedures. These procedures were performed on patients who had been admitted into various hospitals across Germany over 15 years, from 1 January 2006 to 31 December 2020. The enumeration of the coded operations was accomplished by analyzing the dataset designated as 23141-0103. This dataset, which was titled “Operations and Procedures on Fully Inpatient Patients: Germany, Years, Gender, Age Groups, Patient’s Place of Residence, Operations and Procedures”, was provided upon request by the Federal Statistical Office of Germany.

For further data classification, we utilized the German Operation and Procedure Key system (OPS coding system). The surgical procedures under review were divided into arthroscopic and open joint operations (OPS codes: 5-812 for arthroscopic procedures and 5-801 for open joint operations). To analyze the surgical procedures performed on joints of the foot, we examined the OPS codes ending in -k, -m, -n, -p, -q, and -r, which represent the upper ankle joint, lower ankle joint, tarsal joint, tarsometatarsal joint, metatarsophalangeal joint, and toe joint, respectively. Surgical procedures performed on the ankle joint were identified using OPS codes ending in k.

The following three groups were defined: Regeneration group—this group included procedures such as autologous matrix-induced chondrocyte transplantation, cartilage graft removal, in vitro tissue culture implantation, cartilage transplantation, subchondral bone opening with acellular implant insertion, and subchondral bone opening with acellular implant insertion enriched with homologous whole blood or its components. Debridement group—this group encompassed procedures such as the excision of diseased joint cartilage tissue, cartilage debridement, subchondral drilling, and microfracture. Refixation group—procedures in this group involved the refixation of osteochondral fragments and microfracture with fragment fixation. These three groups were formed based on the following OPS codes: regeneration (OPS codes 5-801.[ak-am, bk-br, ck-cr, kk-kr, nk-nr, pk-pr] and 5-812.[8k, 9k-9r, ak-an, gk-gr, hk-hr, mk-mr]), debridement (OPS codes 5-801.[0k-0r, 1k-1r, 2k-2r, gk-gr, hk-hr] and 5-812.[0k-0r, 1k-1r, 2k-2r, 4k-4r, ek-er, fk-fr]), and refixation (OPS codes 5-801.[3k-3r, 4k-4r]; 5-812.[3k-3n, 3r]). The procedures “removal of osteophytes”, “implantation of metallic implant”, and “not specified” were excluded from the analysis. The collected data were subsequently broken down into more detailed subcategories for an in-depth analysis. This was achieved by segregating the data based on various criteria, such as the patient’s age, which was grouped in 5-year intervals for ease of analysis; gender, distinguishing between male and female patients; year of the surgical procedure, encompassing a span from 2006 to 2020; and the localization of the surgery, differentiating between ankle and foot joints.

### 2.1. Data Processing

The dataset 23141-0103 we acquired was essentially a table that encompassed grouped demographic information and OPS codes. It included the following specifics: OPS codes that start with 5-801 and 5-812, age (divided into 22 segments, such as “under 1”, “1–5”, “5–10”, …, “85–90”, “90–95”, “over 95”), gender (divided into two groups: “male” and “female”), and the year of collection (divided into 15 groups: “2006”, “2007”, …, “2018”, “2019”, and “2020”). This data was transferred from a tabular to a list format using the R programming language (courtesy of RStudio PBC; Boston, MA, USA) and the tidyverse package. Furthermore, re-categorization and comprehensive data analysis were carried out using Tableau software (produced by Tableau Software; Seattle, WA, USA).

### 2.2. Statistical Analysis

To demonstrate correlations across time, we employed linear regression analyses, whereas non-linear (Lorentzian) regression analyses were utilized for analyzing the age distribution. All statistical assessments were conducted with the F-test using GraphPad Prism v.9 software (from GraphPad Software; San Diego, CA, USA). Data values are presented in three forms: absolute, relative, and average ± standard deviation. The threshold for statistical significance was established at *p* < 0.05.

## 3. Results

During the study period, 136,501 procedures were analyzed, with a significant majority performed on the ankle (100,055 ankle vs. 36,446 foot; ratio = 2.7:1). Overall, arthroscopic procedures were more common (62,269 arthrotomy vs. 74,232 arthroscopy; ratio = 1:1.2); however, only in the ankle joint, arthroscopic techniques were predominantly used (28,458 arthrotomy vs. 71,597 arthroscopy; ratio = 1:2.5), while open surgery remained prevalent for foot procedures (33,811 arthrotomy vs. 2635 arthroscopy; ratio = 12.8:1) (Table 1).

Distinct gender disparities were observed. Although the overall gender ratio was balanced (67,404 male vs. 69,097 female; ratio = 1:1.02), more ankle procedures were performed on men (56,529 male vs. 43,526 female; ratio = 1:0.77), while women underwent more foot procedures (10,875 male vs. 25,571 female; ratio = 1:2.4) (Table 1).

**Figure 2:**
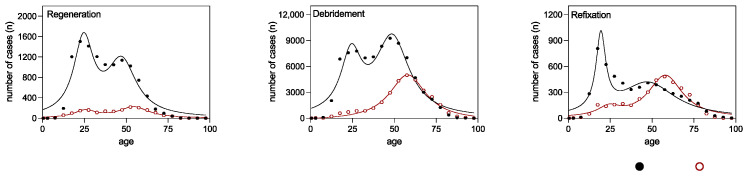
Non-linear regression analysis of ankle (black solid dots) and foot (red hollow dots) surgical procedures from 2006 to 2020, categorized by patient age. Lorentzian distributions are shown for the regenerative, debridement, and refixation OPS codes.

The age distribution exhibited a bimodal pattern for most interventions (Figure 2). The first peak was observed at 25 years of age (calculated age maximum for “regeneration” foot: 25.54 years, ankle: 24.53 years; “debridement” ankle: 24.22 years; “refixation” foot: 24.25 years), except for ankle refixation, where patients were on average 5 years younger (calculated age maximum 19.44 years). The second peak differed between foot and ankle interventions, with ankle patients averaging 48 years old (calculated age maximum for “regeneration”: 47.26 years, “debridement”: 48.74 years, “refixation”: 47.37 years) and foot patients nearly 10 years older (calculated age maximum for “regeneration”: 54.55 years, “debridement”: 57.97 years, “refixation”: 57.93 years). Regenerative therapies of the ankle (“refixation” and “regeneration”) were predominantly performed in younger patients, which was apparent from the clearly higher peak in the age distribution curve, while “palliative” therapies (“debridement”) were more frequent in older patients. The low point between the two age maxima could be regarded as the threshold that could be considered for deciding between regenerative and palliative therapy. This age threshold lay mostly in the age group 35–40 years (calculated age minimum for “regeneration” foot: 40 years, ankle: 36 years; “debridement” foot: 33 years, ankle: 34 years; “refixation” foot: 37 years, ankle: 30 years) (Figure 2). For this reason, the age of 40 years was set as the threshold for further subgroup analysis (Table 2). Foot cartilage surgery was generally performed more often in older age groups.

The majority of surgeries were classified as debridement (n = 114,387), followed by regeneration (n = 13,099) and refixation (n = 9015). Microfracture (n = 58,252) and chondroplasty (n = 56,135) were the most common procedures, followed by the use of acellular membranes (n = 11,414) (Table 2). Microfracture and chondroplasty were more frequent in patients aged over 40 years for both the ankle and foot. Acellular membrane use was preferentially performed in the younger cohort at the ankle. Other cartilage regeneration procedures, such as cartilage transplantation, were less common in total numbers.

**Figure 3:**
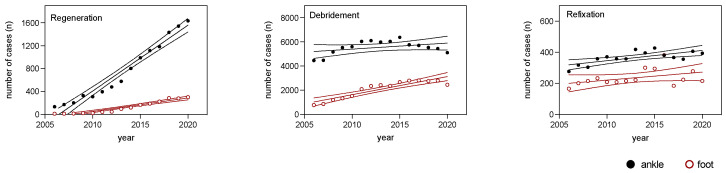
Regression analysis of ankle (black solid dots) and foot (red hollow dots) surgical procedures from 2006 to 2020, showing regression lines and 95% confidence intervals for the regenerative, debridement, and refixation OPS codes.

During the analyzed period, regenerative surgical techniques showed a significantly higher average annual increase in cases for the ankle (115.2 new cases per year) compared with the foot (23.6 new cases per year), with strong correlations (R^2^ = 0.9579 and 0.9256, respectively) and significant increasing slopes. This means that the increase in cases was statistically significant. The number of debridement cases increased annually for both the ankle and foot (ankle: 50.1; foot: 150.4), but only surgical procedures for the foot had a significantly non-zero slope. Refixation for the ankle and foot demonstrated similar numbers of new cases per year (ankle: 6.6; foot: 5.1), with only the ankle having a significantly non-zero slope (6.6; *p* = 0.0033) (Figure 3 and Table 3).

**Figure 4:**
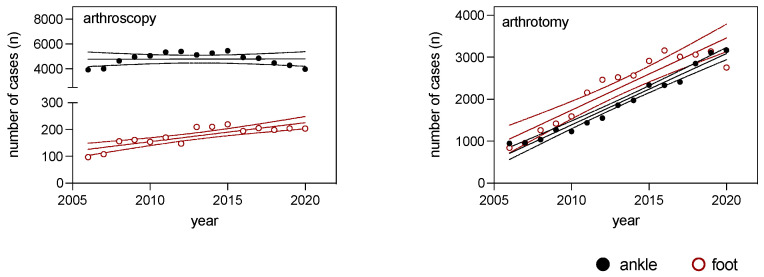
Regression analysis of ankle (black solid dots) and foot (red hollow dots) surgical procedures from 2006 to 2020, displaying regression lines and 95% confidence intervals for arthroscopic and arthrotomic OPS codes.

Arthroscopic surgical procedures of the foot and arthrotomic procedures of both the foot and ankle exhibited significant increases over time (Figure 4 and Table 4). Moreover, no significant differences in the slopes were observed between the ankle and foot arthroscopy procedures. Additionally, no discernible differences were detected between the foot and ankle surgical procedures, encompassing both arthrotomic and arthroscopic techniques (Table 4).

## 4. Discussion

In this study, we demonstrated that differences existed in cartilage surgeries between the foot and ankle joints, both in terms of their epidemiologies and surgical procedures. 

The ankle joint exhibited a higher prevalence of surgery in men, corroborating the general assumption that men are more susceptible to osteochondral lesions in the ankle joint [3]. In contrast, foot surgery patients were more frequently female and older than ankle surgery patients, which may indicate that cartilage lesions in the foot are more often related to arthrosis or deformities, such as hallux valgus. 

Furthermore, the evidence indicated potential disparities in the outcomes of cartilage repair surgery between male and female patients in the context of knee cartilage defects. A recent review underscored the importance of gender differences in cartilage biology and their implications for cartilage degeneration and repair; hence, males and females respond differently to cartilage degeneration and regeneration [10]. The author suggested that anatomical variations in cartilage growth and knee structure between the sexes might play a part in the different rates of cartilage degeneration observed in males and females. The speed of cartilage degradation in a person is influenced by multiple factors, and numerous mechanisms that influence the risk of osteoarthritis and cartilage injury are dependent on the individual’s sex [10]. Moreover, women are more prone to osteoarthritis than men, likely due to estrogen decline post-menopause, implicating estrogen’s role in cartilage degradation and its potential as a treatment for osteoarthritis in women. Conversely, men also suffer from cartilage deterioration and respond to testosterone treatment, suggesting androgen’s role in osteoarthritis. This highlights sex-based variations in hormone therapy responses and treatment options [10]. It is plausible to infer that analogous gender-related differences may be relevant to the foot and ankle given the documented gender-specific differences observed in the foot and ankle anatomies [11] and the similar biologic response of the cartilage upon sex hormone exposure. Consequently, it is crucial to take into consideration these gender-related factors when evaluating the outcomes of cartilage repair surgeries. 

Our study identified two age groups that were particularly affected by cartilage lesions. Experts concur that there is a distinction between “classic” osteochondrosis dissecans in the immature skeleton and osteochondral lesions in adults [1,2], although the etiology of these lesions remains uncertain. Furthermore, the nomenclature has frequently been inconsistent and confusing in the past [1,10].

The different entities of osteochondrosis dissecans and osteochondral lesions explain the bimodal age distribution pattern of our study, with younger patients potentially suffering from osteochondritis dissecans and older patients from osteochondral lesions. Moreover, it elucidates why the patients that underwent refixation of an osteochondral fragment at the ankle were, on average, five years younger than those that received other procedures: Kessler et al. demonstrated the highest incidence of osteochondritis dissecans at the ankle between 12 and 19 years of age (incidence 6.8/100,000) [12]. In accordance with that finding, the age peak for refixation of osteochondral fragments at the ankle in our study was 19.44 years. 

**Figure 5:**
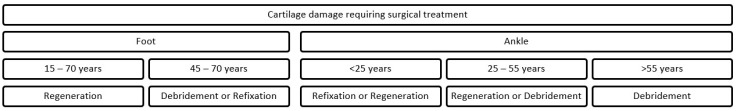
Chart considering the treatments of osteochondral lesions of the foot and ankle from an ex-post perspective considering the age of the patients that underwent surgery. Regeneration includes autologous matrix-induced chondrocyte transplantation, in vitro tissue culture implantation, cartilage transplantation, and subchondral bone opening with acellular implant insertion and its variant enriched with homologous whole blood or its components, while debridement includes cartilage tissue excision, cartilage debridement, subchondral drilling, and microfracture. Refixation involves the reattachment of osteochondral fragments, with or without microfracture.

While cartilage repair interventions are often favored for younger populations, as was also confirmed in this study, evidence shows that age may not significantly impact the results of cartilage repair surgery. Evidence exists that suggests no significant difference in cartilage repair surgery outcomes between younger and older patients. Despite the preference among many surgeons to utilize cartilage repair interventions in younger populations, some studies posited that the chondrogenic potential of mesenchymal stem cells used in cartilage repair is age-independent [13]. A study that assessed the results of a one-stage cartilage repair using a hyaluronic-acid-based scaffold with activated bone-marrow-derived stem cells revealed successful results in patients aged 45 and above, indicating that age does not impede successful cartilage repair [13]. Additionally, a systematic review and meta-analysis of randomized controlled trials that examined the efficacy of culture-expanded mesenchymal stem cells in knee osteoarthritis found no significant disparity in clinical outcomes and cartilage repair between younger and older patients [14]. This indicates that age may not be a decisive factor in the efficacy of cartilage repair using mesenchymal stem cells. Furthermore, an evaluation of the survival rates of various autologous chondrocyte implantation grafts and associated procedures determined that patients aged 40 years and above did not exhibit inferior outcomes up to 24 months following autologous chondrocyte implantation for isolated cartilage defects compared with younger patients [15]. This evidence suggests that age may not substantially impact the outcomes of autologous chondrocyte implantation in older patients. Our data support the increasing utilization of regenerative cartilage repair techniques for treating cartilage defects in the foot and ankle. Although still preferred for treating younger patients, these techniques are often applied to patients over 40, indicating that a patient’s chronological age may not be a limiting factor for cartilage regenerative procedures (Figure 5). The biological age and the activity level should be regarded as more accurate indicators when determining whether to perform regenerative surgery on the foot and ankle.

Concerning intervention types, no validated guidelines exist for treating osteochondral lesions in the ankle or foot. The durability of the microfracture method, especially for larger lesions and for patients with higher activity levels, was met with skepticism [16]. Conversely, the AMIC (autologous matrix-induced chondrogenesis) procedure for talar osteochondral defects exhibited promising clinical and radiological outcomes [17,18]. There has recently been increasing research interest in employing bone marrow aspirate concentrate for cartilage restoration. Animal studies reported promising findings for cartilage regeneration using bone marrow aspirate concentrate; however, additional clinical evidence is needed to determine its safety and efficacy in foot and ankle surgeries [19]. The “International Consensus Meeting of Cartilage Repair of the Ankle” in 2017 sought to address this gap in the guidelines. An expert group offered recommendations for selecting appropriate surgical interventions, focusing primarily on cartilage defect characteristics, such as the size, depth, or presence of cysts [20]. However, no recommendations were provided concerning the patient’s age, which confirms the assumption that the role of age in the selection of the appropriate method remains unclear, even among experts.

Overall, regenerative procedures, especially in the ankle, have exhibited increasing popularity over time, which is potentially attributable to the growing accessibility of novel techniques and materials, such as acellular membranes and in vitro cartilage cultivation. Although these therapies are increasingly employed, the evidence supporting their efficacy remains limited.

To the best of our knowledge, no comparable studies that analyzed cartilage surgery of the foot and ankle from an epidemiological standpoint exist. Therefore, our data could not be compared with data from other countries. It would be interesting to know whether the different surgical procedures are used with the same frequency and distribution in other countries as in Germany, or whether the choice of procedure depends not only on the injury but perhaps also on health policy and monetary decisions.

Our study had several limitations. It exclusively offered an inventory of performed surgical procedures without considering clinical outcomes. Nevertheless, the epidemiological data allowed for inferences regarding the etiology and the effectiveness of various therapies. By employing the Operation Procedure Codes, it is possible that more than one procedure was coded per patient, and repeated interventions on the same patient could not be identified. Consequently, the actual number of patients examined was likely lower than the number of procedures assessed.

## 5. Conclusions

Cartilage surgery involving the foot and ankle is prevalent and demonstrates an increasing trend in Germany. Variations in age and gender were observed concerning the intervention site and procedure type; notably, age played a significant role in determining the choice of surgical therapy.

## Figures and Tables

**Table 1:** Distributions of the procedures, localizations, and sexes of all cases analyzed.

	Σ	Ankle	Foot
Procedure			
Arthrotomy	62,269	28,458	33,811
Arthroscopy	74,232	71,597	2635
Sex			
Male	67,404	56,529	10,875
Female	69,097	43,526	25,571

Summary of the total number of surgical procedures (Σ) performed between 2006 and 2020, categorized by localization (ankle and foot), procedure type (arthrotomy and arthroscopy), and patient sex (male and female).

**Table 2:** Surgical procedures performed on the cartilage of the ankle and foot between 2006 and 2020, categorized by patients younger or older than 40 years of age.

Procedure	Σ	Ankle		Foot	
		≤39 Years	≥40 Years	≤39 Years	≥40 Years
Debridement					
Chondroplasty, excision of diseased cartilage	56,135	19,342	24,805	2011	9977
Microfracture, subchondral drilling	58,252	19,086	20,055	2425	16,686
Σ	114,387	38,428	44,860	6447	26,663
Regeneration					
Autologous matrix-induced chondrocyte transplantation, microfracture with application of a membrane	11,414	5593	4136	673	1012
Cartilage transplantation, cartilage graft harvesting, in vitro tissue culture implantation	1685	986	573	51	75
Σ	13,099	6579	4709	724	1087
Refixation					
Σ	9015	2951	2528	829	2707

**Table 3:** Linear regression analysis for the surgical procedures of the ankle and foot from 2006 to 2020 (complementary table to Figure 2). Values are given for the regeneration, debridement, and refixation groups (slope, intercept on the *y*-axis, R^2^, and level of significance) for simple regression lines.

	Average New Cases Per Year (Slope of the Regression Line)	Intercept of the Regression Line	R^2^	Is Each Slope Significantly Non-Zero?	Are the Differences between the Slopes (Ankle vs. Foot) Significant?
Regeneration					
Ankle	115.2	−231,217	0.9579	Yes (*p* < 0.0001)	Yes (F = 173.7; *p* < 0.0001)
Foot	23.6	−47,393	0.9256	Yes (*p* < 0.0001)	
Debridement					
Ankle	50.1	−95,277	0.1583	No (*p* = 0.1419)	Yes (F = 7.259; *p* = 0.0122)
Foot	150.4	−300,668	0.8287	Yes (*p* < 0.0001)	
Refixation					
Ankle	6.6	−12,942	0.4974	Yes (*p* = 0.0033)	No (F = 0.1626; *p* = 0.6901)
Foot	5.1	−10,138	0.1745	No (*p* = 0.1212)	

**Table 4:** Linear regression analysis for the surgical procedures of the ankle and foot from 2006 to 2020 (complementary table to Figure 3). Values are given for the arthrotomy and arthroscopy groups (slope, intercept on the y-axis, R^2^, and level of significance) for simple regression lines.

	Average New Cases Per Year (Slope of the Regression Line)	Intercept of the Regression Line	R^2^	Is Each Slope Significantly Non-Zero?	Are the Differences between the Slopes (Ankle vs. Foot) Significant?
Arthroscopy					
Ankle	1.9	883	0.0001	No (*p* = 0.9544)	No (F = 0.02; *p* = 0.8776)
Foot	7.1	−14,095	0.6999	Yes (*p* < 0.0001)	
Arthrotomy					
Ankle	170.0	−340,320	0.9708	Yes (*p* < 0.0001)	No (F = 0.01; *p* = 0.9199)
Foot	172.1	−344,104	0.8691	Yes (*p* < 0.0001)	

## Data Availability

The datasets generated during the current study are available from the corresponding author upon reasonable request.
